# GNA13 regulates BCL2 expression and the sensitivity of GCB-DLBCL cells to BCL2 inhibitors in a palmitoylation-dependent manner

**DOI:** 10.1038/s41419-020-03311-1

**Published:** 2021-01-09

**Authors:** Zhizhou Xia, Xiuli Zhang, Ping Liu, Ruihong Zhang, Zhangsen Huang, Donghe Li, Xinhua Xiao, Min Wu, Nannan Ning, Qianqian Zhang, Jianmin Zhang, Mingzhu Liu, Bo Jiao, Ruibao Ren

**Affiliations:** Shanghai Institute of Hematology, State Key Laboratory for Medical Genomics, Collaborative Innovation Center of Hematology, National Research Center for Translational Medicine, Ruijin Hospital affiliated to Shanghai Jiao Tong University School of Medicine, Shanghai, China

**Keywords:** B-cell lymphoma, B-cell lymphoma, Translational research

## Abstract

*GNA13*, encoding one of the G protein alpha subunits of heterotrimeric G proteins that transduce signals of G protein-coupled receptors (GPCR), is frequently mutated in germinal center B-cell-like diffuse large B-cell lymphoma (GCB-DLBCL) with poor prognostic outcomes. Due to the “undruggable” nature of GNA13, targeted therapy for these patients is not available. In this study, we found that palmitoylation of GNA13 not only regulates its plasma membrane localization, but also regulates GNA13’s stability. It is essential for the tumor suppressor function of GNA13 in GCB-DLBCL cells. Interestingly, GNA13 negatively regulates BCL2 expression in GCB-DLBCL cells in a palmitoylation-dependent manner. Consistently, BCL2 inhibitors were found to be effective in killing GNA13-deficient GCB-DLBCL cells in a cell-based chemical screen. Furthermore, we demonstrate that inactivating GNA13 by targeting its palmitoylation enhanced the sensitivity of GCB-DLBCL to the BCL2 inhibitor. These studies indicate that the loss-of-function mutation of GNA13 is a biomarker for BCL2 inhibitor therapy of GCB-DLBCL and that GNA13 palmitoylation is a potential target for combination therapy with BCL2 inhibitors to treat GCB-DLBCL with wild-type GNA13.

## Introduction

*GNA13* encodes one of the alpha subunits (GNA13/Gα13) of the heterotrimeric G proteins that transduce signals of G protein-coupled receptors (GPCR). It is expressed in various tissues, including lymphoid, vascular, and bone tissues in embryos and adults. Although GNA13 is classified into the Gα12/13 subfamily and highly homologous to GNA12^1^, GNA13 has unique functions. It has been shown to play critical roles in localization of germinal center (GC) B cells^2^, angiogenesis^3^, female fertility^4,5^, bone homeostasis^6^, and platelet activation^7,8^.

Recurrent mutations in the *GNA13* gene have been identified in multiple tumor types. As GNA13 activation can promote migration, invasion, and metastasis in pancreas, prostate, and ovarian cancer, it was originally classified as an oncogene^9–11^. However, loss-of-function mutations in *GNA13* have recently been identified in diffuse large B-cell lymphoma (DLBCL)^12–14^, indicating that GNA13 may also function as a tumor suppressor. Consistent with this observation, GNA13-deficient mice develop GC B-cell-derived lymphoma^2^.

DLBCL is the most commonly diagnosed lymphoma and accounts for 25–35% of all B-cell non-Hodgkin lymphomas^15^. Based on the gene expression pattern and cell-of-origin, DLBCL is usually classified into two main subtypes, namely, GC B-cell-like (GCB) and activated B-cell-like (ABC) DLBCL^16,17^. Although nearly 60% of DLBCL patients can be cured by Rituximab plus chemotherapy-based standard treatment (R-CHOP), the rest may die due to therapy nonresponsiveness or disease relapse resulting from the complexity and heterogeneity of the disease^13^. Identifying valuable therapeutic targets for treating DLBCL remains an urgent need.

In the GC, B cells are strictly confined within follicles by the GPCR signaling, such as sphingosine-1-phosphate receptor S1PR2 and purinergic receptor P2RY8 signaling^18–20^. GNA13 was found to activate ARHGEF1-RHOA and subsequently inhibits the phosphoinositide 3-kinase (PI3K)/AKT pathway^21^. A recent CRISPR/Cas9-based screen in primary GC B cells showed that GNA13 depletion strikingly enhances cell survival and proliferation, indicating its major suppressive role in constraining GC B cells^22^. Consistent with this, over 18% of germinal center B-cell-like diffuse large B-cell lymphoma (GCB-DLBCL) patients harbor loss-of-function mutations or homozygous deletions in the *GNA13* gene locus^12–14^. Additionally, some partners of *GNA13*, like *S1PR2*, *P2RY8*, *ARHGEF1*, and *RHOA*, are also frequently mutated or dysregulated in GCB-DLBCL, implying the critical role of this GPCR signaling in lymphomagenesis^12,23,24^.

Although GCB-DLBCL prognosis is generally more favorable than that of ABC-DLBCL, a recent comprehensive analysis of 1001 DLBCL patients revealed that GCB-DLBCL patients who harbor *GNA13* mutations and also express high level of *BCL2* have an extraordinarily high risk of poor outcomes^25^. However, no effective therapeutic strategy is available for this DLBCL subtype.

Post-translational protein modifications regulate protein function and can be used as therapeutic targets. S-palmitoylation involves palmitoyl acyltransferase (PAT)-mediated covalent lipid modification of cysteine side chains with the 16-carbon fatty acid, palmitate^26,27^. Palmitoylation regulates the membrane association, subcellular trafficking, stability, and function of proteins^26^. We previously showed that palmitoylation of NRAS is essential for its plasma membrane (PM) translocation, signal transduction, and leukemogenesis, both in vivo and in vitro^28^.

Palmitoylation is required for GNA13 to associate with the PM and the activation of Rho-dependent signaling^29^. Here, we show that palmitoylation of GNA13 also regulates its stability and is required for its tumor suppressor function in GCB-DLBCL cells. Interestingly, GNA13 negatively regulated BCL2 expression in GCB-DLBCL cells in a palmitoylation-dependent manner. Inactivating GNA13 by targeting its palmitoylation enhanced the sensitivity of GCB-DLBCL cells to the BCL2 inhibitors. Our studies suggested that GNA13 loss-of-function mutations may serve as a biomarker for BCL2 inhibitor-mediated precision therapy of DLBCL and that GNA13 palmitoylation may be a potential target for combination therapy with BCL2 inhibitors to treat DLBCL with wild-type (WT) GNA13.

## Results

### Palmitoylation regulates GNA13 protein stability

**Fig. 1:**
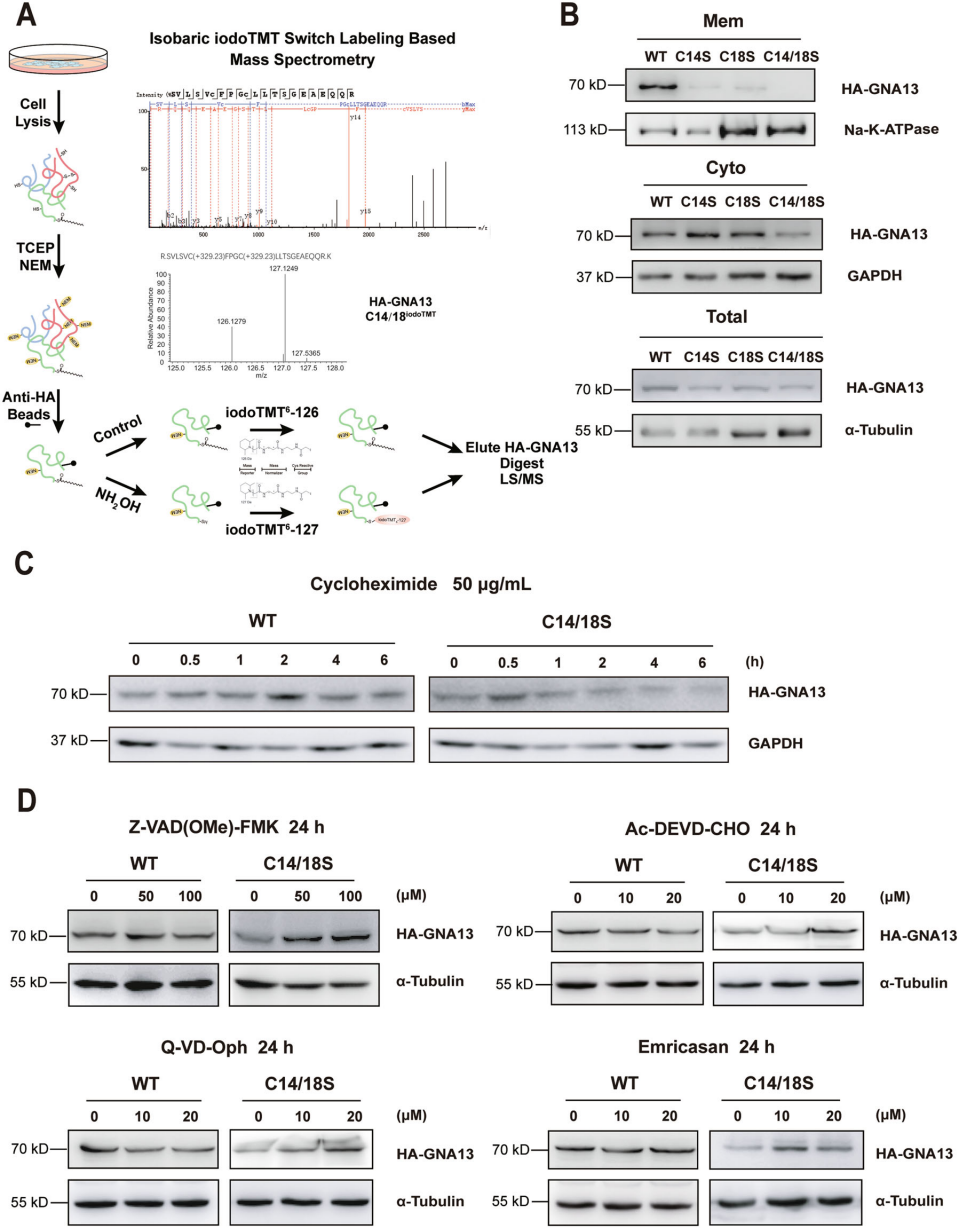
Palmitoylation of GNA13 regulates its protein stability. **A** The scheme of isobaric iodoTMT switch labeling-based mass spectrometry assay and results of MS/MS spectrum of palmitoylated peptide of GNA13. IodoTMT^6^-127 labeling on Cys14 and Cys18 (the two C in lowercase) of GNA13 peptide sequence was shown in peaks graph (upper panel). **B** Total, membrane (Mem) and cytosolic (Cyto) fractions of HeLa cells expressing HA-tagged WT GNA13, C14S, C18S, or C14/18S mutant of GNA13 were immunoblotted with an anti-HA antibody. α-Tubulin was used as a loading control for total cellular proteins, while Na-K-ATPase and GAPDH were used as markers of the membrane and cytosol, respectively. **C** HeLa cells overexpressing WT GNA13 or C14/18S mutant were incubated with cycloheximide (CHX) and analyzed by western blot at the indicated time points. **D** Protein levels of WT GNA13 and C14/18S mutant in HeLa cells treated with or without indicated caspase inhibitors for 24 h were detected by immunoblotting with an anti-HA antibody. α-Tubulin was used as a loading control.

To elucidate the role of GNA13 palmitoylation in GCB-DLBCL, we first confirmed the palmitoylation sites in GNA13 employing isobaric iodoTMT switch labeling in HeLa cells stably expressing HA-tagged GNA13. The proteomics data showed that both cysteine 14 (C14) and 18 (C18) contained iodoTMT^6^-127, indicative of palmitoyl modifications (Fig. 1A). All other cysteines could be excluded as palmitoylation sites except for C236, because the tryptic peptide containing this residue could not be resolved by mass spectrometry owing to its small size. Similarly, a click chemistry-based, single-cell in situ proximity ligation assay (Supplementary Fig. S1A–C) showed that GNA13 was palmitoylated (red fluorescence) and that palmitoylation was almost abolished by the C14/18S double mutation. We further confirmed the above results using bioinformatic algorithms (CSS-PALM 4.0^30^, MDD-PALM^31^) and an Acyl-RAC assay (Supplementary Fig. S1D, E). These results were consistent with previous findings^29^.

Next, we characterized the PM localization of GNA13 by co-staining with the PM marker, Na-K ATPase (Supplementary Fig. S3A). A large fraction of WT GNA13 (GNA13^WT^) localized to the PM, whereas GNA13 harboring the single or double mutations in palmitoylation sites showed diffused cytoplasmic staining. Furthermore, 2-bromopalmitate, a pan-palmitoylation inhibitor, markedly inhibited the membrane localization of GFP-GNA13^WT^ after a short treatment (Supplementary Fig. S3B). We further employed a biochemical method to isolate the PM and cytosolic fractions of the cell. Consistent with a previous report^29^, the data showed that the PM fraction of palmitoylation-deficient GNA13 mutants was considerably less than that of GNA13^WT^ (Fig. 1B).

Notably, the expression level of the GNA13^C14/18S^ mutant was lower than that of GNA13^WT^, as well as that of the single mutants (Fig. 1B), indicating that palmitoylation affected protein stability. A cycloheximide-based pulse-chase experiment revealed that the loss of palmitoylation accelerated GNA13 degradation compared with GNA13^WT^ (Fig. 1C).

Palmitoylation can regulate protein stability by affecting critical proteolytic processes, such as those associated with the ubiquitin-proteasome^32,33^, autophagosome-lysosome^34^, and caspase systems^35,36^. To examine if palmitoylation regulates GNA13 protein stability through these systems, we tested whether the downregulation of the GNA13^C14/18S^ mutant could be rescued by treatment with the proteasomal inhibitor MG132, the autophagy inhibitors HCQ and ULK-101, and several caspase inhibitors. Interestingly, the downregulation of GNA13^C14/18S^ was marked reversed by exposure to the pan-caspase inhibitors Z-VAD(OMe)-FMK, Q-VD-Oph, and Emricasan, as well as by the group II caspase-specific inhibitor Ac-DEVD-CHO (Fig. 1D), indicating that the palmitoylation of GNA13 regulates its stability through the evasion of caspase-associated degradation.

Interestingly, the level of GNA13^WT^ can be upregulated by MG-132 (Supplementary Fig. S2C), indicating that GNA13 is also subject to proteasome-mediated degradation. Meanwhile, protein levels of both GNA13^WT^ and GNA13^C14/18S^ can be moderately upregulated by autophagic inhibitors HCQ and ULK-101 (Supplementary Fig. S2D), suggesting that GNA13 can also be regulated by the autophagosome-lysosome system. Palmitoylation does not appear to affect these processes.

These data demonstrated that GNA13 palmitoylation regulates both its PM localization and its stability.

### Palmitoylation of GNA13 is required for its tumor suppressor function

GNA13 is frequently mutated in GCB-DLBCL. Various sites are mutated throughout the *GNA13* gene, consistent with their loss-of-function nature^11–14^. Through the whole genome sequencing data obtained from St. Jude Cloud^37^, we found that there were at least two patients harboring the GNA13 C14S mutation (Supplementary Fig. S3)^38,39^, suggesting that palmitoylation of GNA13 regulates its tumor suppression function.

**Fig. 2:**
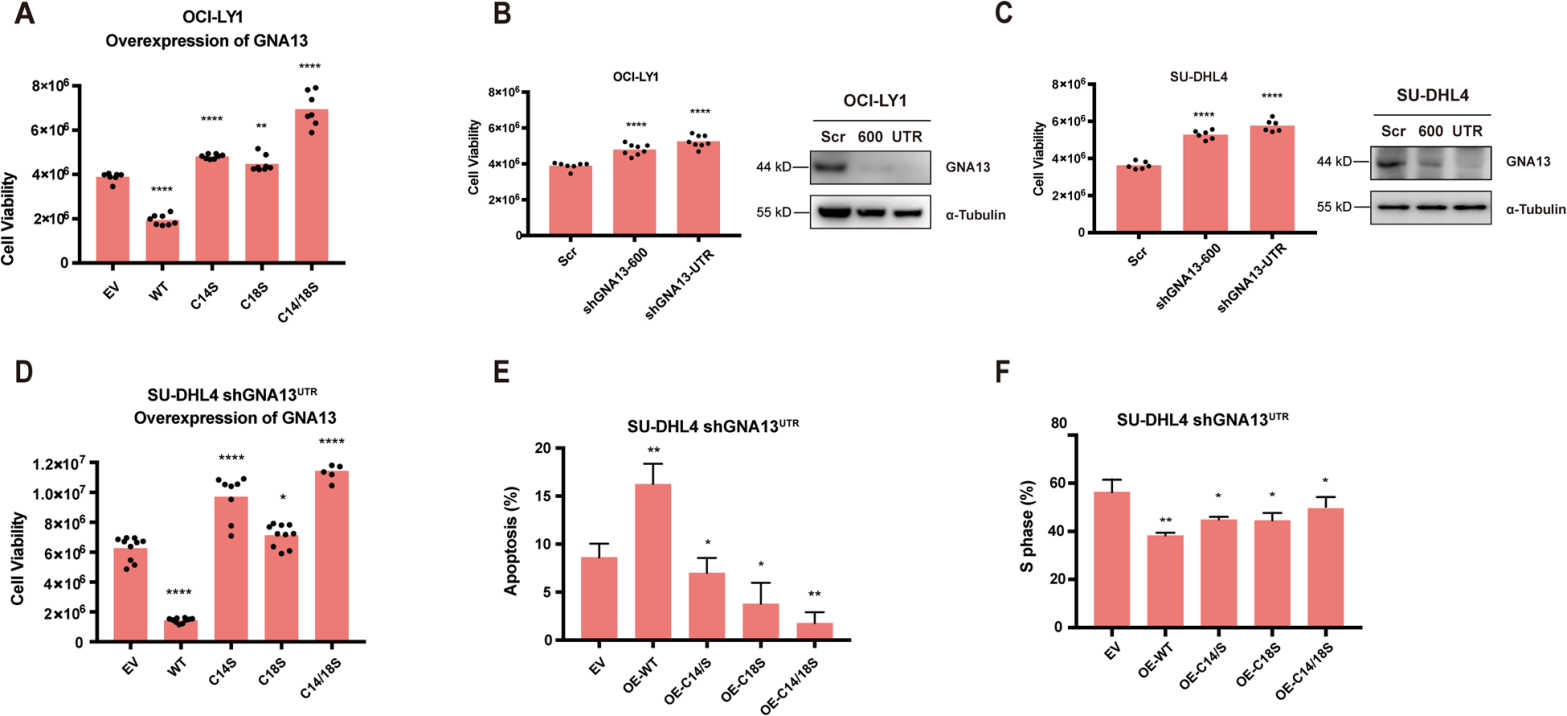
Palmitoylation of GNA13 is required for its tumor suppressor function. **A** Viability of OCI-LY1 cells transfected with empty vector (EV), WT, C14S, C18S, or C14/18S *GNA13* construct measured by CellTiter-Glo Luminescent Cell Viability Assay 48 h after plating. **B**, **C** Knocking down of the endogenous *GNA13* in OCI-LY1 (**B**) or SU-DHL4 (**C**) cells by two GNA13-specific shRNAs, shGNA13-600 and shGNA13-UTR. Scrambled shRNA (Scr) was used as the negative control. Cell viability of each cell line was measured by CellTiter-Glo Luminescent Cell Viability Assay 48 h after plating. Protein levels of GNA13 in these cells were examined by western blot analysis. α-Tubulin was used as loading controls. **D** SU-DHL4 cells expressing shGNA13-UTR were transfected with EV, HA-tagged WT, C14S, C18S, or C14/18S mutant *GNA13*. Cell viability of each cell line was measured by CellTiter-Glo Luminescent Cell Viability Assay 48 h after plating. **E** Apoptosis assay of each SU-DHL4 cell line using Annexin V/PI staining was examined by flowcytometry 48 h after plating. OE, overexpression. **F** Cell-cycle analysis of each SU-DHL4 cell line by using BrdU/7-AAD staining. Percentage of the cells at S phase was shown as the proliferative portion of whole population.

To examine the role of palmitoylation in GNA13’s tumor suppressor function, we compared the tumor suppressor activity of palmitoylation-deficient mutants of GNA13 to that of WT counterpart in two GCB-DLBCL cell lines. First, we transduced GNA13^WT^, GNA13^C14S^, GNA13^C18S^, and GNA13^C14/18S^ into OCI-LY1, a GCB-DLBCL cell line harboring loss-of-function *GNA13* mutations. Consistent with the tumor suppressor function of GNA13, we found that ectopic expression of GNA13^WT^ markedly suppressed proliferation of OCI-LY1 cells (Fig. 2A). The three GNA13 palmitoylation mutants, on the other hand, did not inhibit proliferation of OCI-LY1 cells (Fig. 2A), indicating that palmitoylation of GNA13 is required for its tumor suppressor function.

Interestingly, the palmitoylation mutants of GNA13, particularly GNA13^C14/18S^, actually promoted proliferation of OCI-LY1 cells (Fig. 2A). This result suggests that OCI-LY1 cells retain partial GNA13 tumor suppressor functions, either the GNA13 mutant in OCI-LY1 cells retain partial tumor suppressor function or the presence of the wild-type allele of GNA13, and that palmitoylation mutants of GNA13 have a dominant negative effect. To test this possibility, we knocked down the endogenous GNA13 by introducing GNA13 shRNAs (Fig. 2B). We found that two independent *GNA13* shRNAs, shGNA13^600^ and shGNA13^UTR^, both promoted proliferation of OCI-LY1 cells (Fig. 2B), supporting the assumption that OCI-LY1 cells retain partial GNA13 tumor suppressor functions.

Next, we further confirm the role of palmitoylation in GNA13’s tumor suppressor function in SU-DHL4, a GCB-DLBCL cell line with the WT *GNA13*. In this experiment, we first transfected SU-DHL4 cells with either scrambled or GNA13-specific shRNAs. The endogenous GNA13 expression was significantly reduced in SU-DHL4 cells transfected with specific shRNAs (Fig. 2C). More importantly, proliferation of SU-DHL4 cells transfected with *GNA13* shRNAs was significantly increased as compared to SU-DHL4 cells transfected with control shRNA (Fig. 2C). We chose SU-DHL4 cell line stably expressing the *GNA13*-UTR shRNA (SU-DHL4-shGNA13^UTR^) for further experiments.

We then transduced GNA13^WT^, GNA13^C14S^, GNA13^C18S^, and GNA13^C14/18S^ into SU-DHL4-shGNA13^UTR^ cells. As expected, ectopic expression of GNA13^WT^ markedly suppressed proliferation of SU-DHL4-shGNA13^UTR^ cells (Fig. 2D). The three GNA13 palmitoylation mutants, on the contrary, did not inhibit proliferation of SU-DHL4-shGNA13^UTR^ (Fig. 2D), further demonstrating that palmitoylation of GNA13 is required for its tumor suppressor function. The increased proliferation of SU-DHL4-shGNA13^UTR^ expressing palmitoylation mutants of GNA13 compared to that transfected with the vector control may be a result of the dominant negative effect of palmitoylation mutants of GNA13 over the residual GNA13, as discussed above (Fig. 2A, D).

In addition, we found that SU-DHL4-shGNA13^UTR^ expressing palmitoylation mutants of GNA13 exhibited a significant decrease in annexin V/PI positive apoptotic cell population compared to SU-DHL4-shGNA13^UTR^ expressing the WT GNA13 (Fig. 2E and Supplementary Fig. S4A), indicating that palmitoylation of GNA13 is required for its pro-apoptotic function. We also found that the cells overexpressing GNA13^WT^ showed a higher level of the cleaved Caspase3, which means the WT GNA13 could induce an active state of caspase3 (Supplementary Fig. S5). Consistently, a similar trend in cell proliferation could also been observed among these cells when we assessed the cell-cycle progression using a BrdU/7-AAD labeling assay (Fig. 2F and Supplementary Fig. S4B).

### GNA13 negatively regulates BCL2 expression in GCB-DLBCL in a palmitoylation-dependent manner

**Fig. 3:**
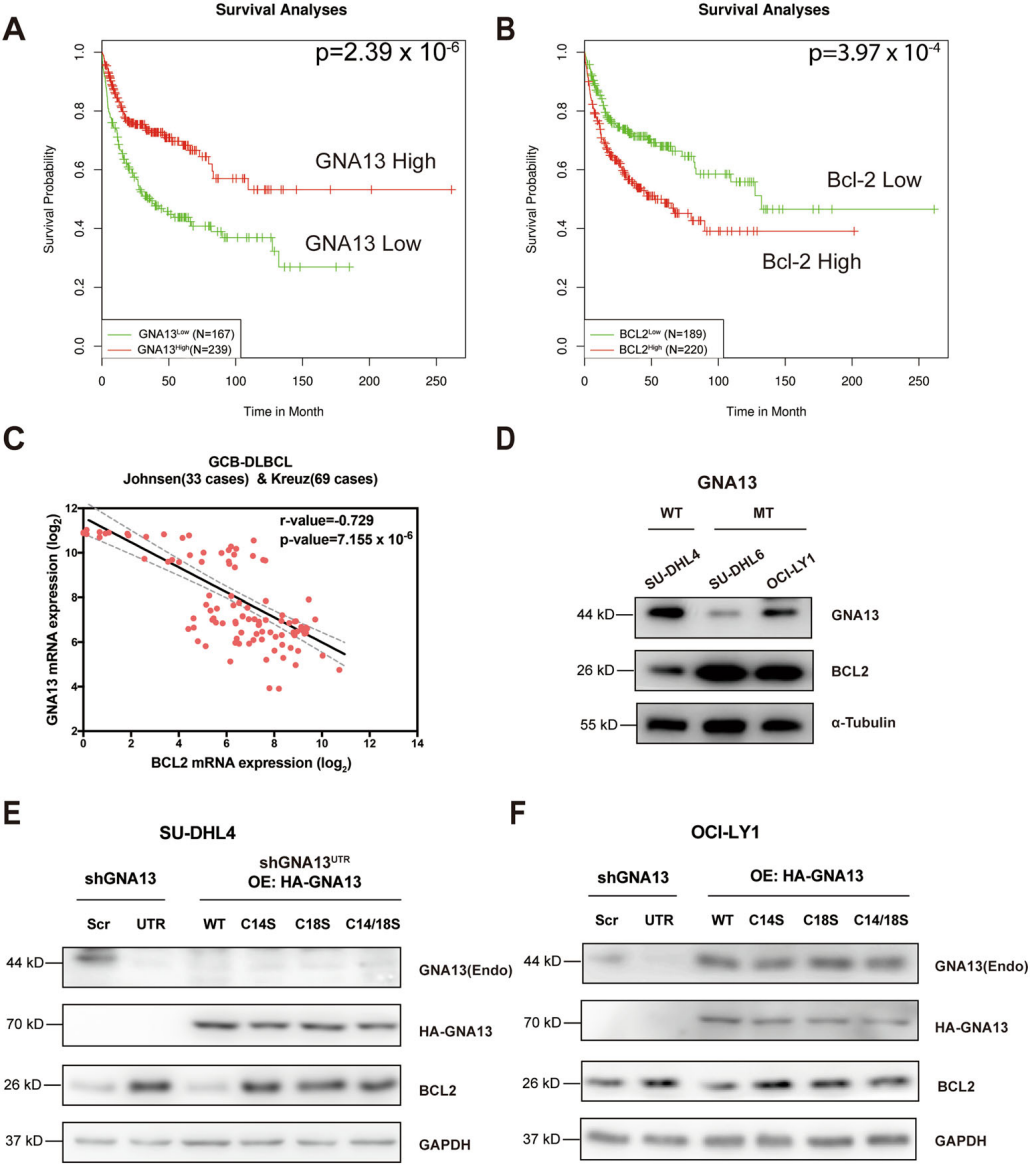
GNA13 regulates the BCL2 expression in GCB-DLBCL cells in a palmitoylation-dependent manner. **A**, **B** Relationship between survival of GCB-DLBCL and expression levels of *GNA13* and *BCL-2* was analyzed based on public datasets (PPISURV). (**A**) High-level expression of *GNA13* is significantly associated with positive outcome of GCB-DLBCL. (**B**) Overexpression of *BCL-2* is significantly associated with negative outcome of GCB-DLBCL. **C** Plot of *GNA13* expression vs. *BCL2* expression in 102 GCB-DLBCL cases (R2: Genomics Analysis and Visualization Platform, https://hgserver1.amc.nl/cgi-bin/r2/main.cgi). The transcript levels of *GNA13* and *BCL2* are inversely related (*r* = −0.729, *P* = 7.155 × 10^−6^). **D** Western blot analysis of GNA13 and BCL2 expressions in SU-DHL4 cells, which harbor the WT GNA13, OCI-LY1, and SU-DHL6 cells, both of which harbor mutant GNA13. α-Tubulin was used as a loading control. **E** Western blot analysis of GNA13 and BCL2 expressions in SU-DHL4 cells expressing scrambled shRNA (Scr) or *GNA13* knockdown shRNA (UTR) (left two lanes), and the SU-DHL4-shGNA13^UTR^ cells ectopically overexpressing (OE) HA-tagged WT, C14S, C18S, or C14/18S mutant GNA13 (right four lanes). GAPDH was used as a loading control. **F** Western blot analysis of GNA13 and BCL2 expressions in OCI-LY1 cells expressing scrambled shRNA (Scr) or *GNA13* knockdown shRNA (UTR) (left two lanes) and the OCI-LY1 cells ectopically expressing HA-tagged WT, C14S, C18S, or C14/18S mutant GNA13 (right four lanes). GAPDH was used as a loading control.

Intriguingly, the clinical data from PPISURV^40^ revealed that *GNA13* and *BCL2* expressions exhibited opposite prognostic effects on the survival of DLBCL patients (Fig. 3A, B). To gain insights into the mechanism by which GNA13 functions as a tumor suppressor in GCB-DLBCL, we analyzed RNA sequencing data of 102 GCB-DLBCL cases from public datasets (R2: Genomics Analysis and Visualization Platform, https://hgserver1.amc.nl/cgi-bin/r2/main.cgi). We found that *BCL2* expression and *GNA13* expression are inversely correlated (Fig. 3C).

To check if the BCL2 expression is affected by the GNA13 activity, we examined BCL2 expression levels in GCB-DLBCL cell lines either with the WT GNA13 (SU-DHL4) or mutant GNA13 (OCI-LY1 and SU-DHL6). We found that the expression of BCL2 is significantly higher in cells with GNA13 mutant than the cells with WT GNA13 (Fig. 3D). These data suggest that GNA13 may exert its tumor suppressor function partially by regulating the BCL2 expression.

To test this hypothesis, we examined the expression level of BCL2 in SU-DHL4 cells vs. SU-DHL4-shGNA13^UTR^ cells. We found that BCL2 expression was drastically elevated when GNA13 was knocked down (Fig. 3E), indicating that GNA13 negatively regulates the expression of BCL2. We then moved on to do a rescue experiment and found that ectopic expression WT GNA13 in SU-DHL4-shGNA13^UTR^ inhibited the expression of BCL2 (Fig. 3E), confirming that GNA13 negatively regulates BCL2 expression level.

Consistent with the previous finding that palmitoylation of GNA13 is required for its tumor suppressor function, ectopic expression of palmitoylation mutants of GNA13 were found to be incapable of suppressing the expression of BCL2 in SU-DHL4-shGNA13^UTR^ cells (Fig. 3E). Similar results were obtained in OCI-LY1 cells bearing a loss-of-function mutant of *GNA13* (Fig. 3F). These data demonstrate that GNA13 is a negative regulator of BCL2 and that palmitoylation is required for this function of GNA13.

### GNA13-deficient GCB-DLBCL cells are hypersensitive to the treatment with BCL2 inhibitors

**Fig. 4:**
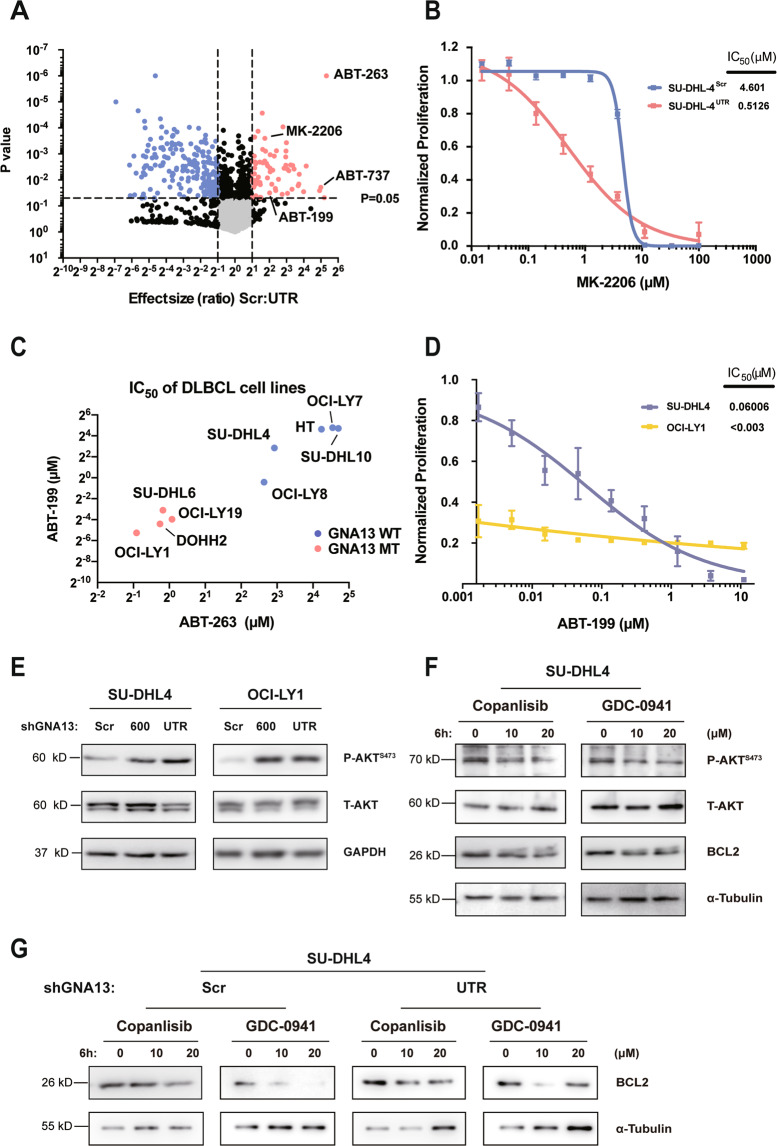
*GNA13-*deficient GCB-DLBCL cells are hypersensitive to BCL2 inhibitors. **A** Volcano plot of FDA-approved drugs and bioactive compounds with known targets on SU-DHL4-shGNA13^Scr^ and SU-DHL4-shGNA13^UTR^ cells. Drug effect size ratio between SU-DHL4-shGNA13^Scr^ and SU-DHL4-shGNA13^UTR^ vs. statistical significance (*P* value) were plotted. Red and blue points indicate drug identified as differentially inhibited between the two types of cells. **B** Dose–response curves for SU-DHL4-shGNA13^Scr^ or SU-DHL4-shGNA13^UTR^ treated with AKT inhibitor MK-2206. **C** IC_50_ of BCL2 inhibitors ABT-199 and ABT-263 in DLBCL cell lines with either wild-type (blue dots) or mutant (red dots) *GNA13*. **D** Dose–response curves for SU-DHL4 and OCI-LY1 cells treated with ABT-199. **E** SU-DHL4 or OCI-LY1 cells were transfected with constructs containing shGNA13-600, shGNA13-UTR, or a scrambled shRNA (Scr). Levels of total (T)-AKT and phosphorylated (P)-AKT^S473^ in these cells were examined by western blot analysis. GAPDH was used as loading controls. **F** Western blot analysis of BCL2, total (T)-AKT, and phosphorylated (P)-AKT^S473^ level in SU-DHL4 cells treated with PI3K inhibitors Copanlisib or GDC-0941 at indicated concentrations for 6 h, respectively. **G** Western blot analysis of BCL2 level in SU-DHL4-shGNA13^Scr^ or SU-DHL4-shGNA13^UTR^ cells treated with PI3K inhibitors Copanlisib or GDC-0941 at indicated concentrations for 6 h, respectively. α-Tubulin was used as a loading control.

To find potential therapies for GNA13-deficient GCB-DLBCL, we carried out a cell-based drug screening using a chemical library comprising FDA-approved drugs and bioactive compounds with known targets. The SU-DHL4-shGNA13^UTR^ described above was used as a model for GNA13-deficient GCB-DLBCL and the parental SU-DHL4 was used for the counter screening. We found that two BCL2 inhibitors, ABT-737 and ABT-263 (the first- and second-generation BCL2 inhibitors, respectively), exhibited the most significant efficacy in killing SU-DHL4-shGNA13^UTR^ as compared to the SU-DHL4 control cells (Fig. 4A).

It has been shown by us (Fig. 4E) and others^2^ that the PI3K-AKT signaling pathway is a well-known downstream target of GNA13. We found that inhibitors of the PI3K-AKT signaling pathway, such as MK-2206, can also effectively kill GNA13-deficient SU-DHL4 cells (Fig. 4A, B), demonstrating the validity of this screening.

Having found that GNA13-deficient SU-DHL4 cells are hypersensitive to the treatment with BCL2 inhibitors, we analyzed sensitivity of DLBCL cell lines to BCL2 inhibitors from published data^41^. Figure 4C shows that DLBCL cell lines with GNA13 mutations are more sensitive to the treatment with BCL2 inhibitors than those with WT GNA13. To further confirm that GCB-DLBCL cells with GNA13 mutations are hypersensitive to the treatment with BCL2 inhibitors, we compared the sensitivity of ABT-199 (also known as venetoclax, the third-generation BCL2 inhibitor^41^) in treating GCB-DLBCL cell lines either with the WT GNA13 (SU-DHL4) or mutant GNA13 (OCI-LY1). As shown in Fig. 4D, OCI-LY1 cells are much more susceptible to the treatment with ABT-199 than SU-DHL4 cells. Consistent with the function of GNA13 in suppressing the phosphoinositide 3-kinase (PI3K)/AKT pathway^21^, the AKT Serine 473 phosphorylation (P-AKT^S473^) level was elevated in SU-DHL4 cells transfected with *GNA13* shRNAs (Fig. 4E). To test whether the high expression of BCL2 is correlated to the activation of PI3K-AKT pathway in DLBCL, we used the pan-PI3K inhibitor GDC-0941 and PI3Kα/δ inhibitor Copanlisib^42^ in treating WT SU-DHL4 cells. Both of the inhibitors could suppress the phosphorylation of AKT^S473^ and BCL2 protein expression accordingly (Fig. 4F). Likewise, in SU-DHL4-shGNA13^UTR^ cells, the protein expression level of BCL2 was also drastically repressed upon PI3K-AKT pathway inhibition by the above two inhibitors (Fig. 4G), implying a high correlation between PI3K-AKT signaling and BCL2 anti-apoptosis pathway may exist in the GNA13-deficient background.

These data, together with the above finding that GNA13 negatively regulates the expression of BCL2, suggest that the loss-of-function mutation of GNA13 is a biomarker for the precision BCL2 inhibitor therapy for GCB-DLBCL.

### Inactivation of GNA13 by targeting its palmitoylation sensitizes the GCB-DLBCL cells to BCL2 inhibitors

Although GCB-DLBCL with loss-of-function mutation of GNA13 could be treated effectively with BCL2 inhibitors, the majority of GCB-DLBCL patients harbor WT GNA13. As GNA13 negatively regulates BCL2 expression, the sensitivity of GCB-DLBCL to the treatment with BCL2 inhibitors, the effective therapy of GCB-DLBCL with WT GNA13 may be achieved by targeting both GNA13 and BCL2. Since our data show that palmitoylation of GNA13 is required for its function in regulating the BCL2 expression, inhibiting GNA13 palmitoylation may sensitize the GCB-DLBCL cells with WT GNA13 to BCL2 inhibitors.

**Fig. 5:**
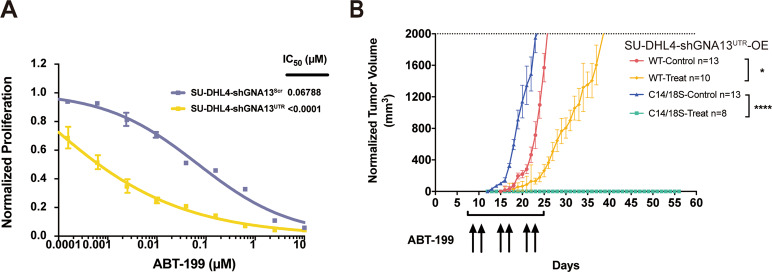
GCB-DLBCL cells with palmitoylation-deficient GNA13 are hypersensitive to BCL2 inhibitor. **A** Dose–response curves for SU-DHL4-shGNA13^Scr^ and SU-DHL4-shGNA13^UTR^ cells treated with ABT-199. **B** Tumor growth curves of SU-DHL4-shGNA13^UTR^ cells either expressing the WT GNA13 (SU-DHL4-shGNA13^UTR^-OE^WT^) or the C14/C18S palmitoylation mutant of GNA13 (SU-DHL4-shGNA13^UTR^-OE^C14/18S^) in mice treated with either BCL2 inhibitor ABT-199 or vehicle. *n*, number.

As a proof-of-concept experiment, we attempted to test the sensitivity of GCB-DLBCL cells bearing palmitoylation mutant of GNA13 to the treatment with BCL2 inhibitor. We first confirmed that SU-DHL4-shGNA13^UTR^ cells, in which the WT *GNA13* was knocked down with the specific GNA13 shRNA as described earlier, were more susceptible to the treatment with ABT-199 compared to the parental SU-DHL4 cells (Fig. 5A).

To test the ABT-199 efficacy in vivo, we generated two xenograft models by serially transplanting SU-DHL4-shGNA13^UTR^-OE^WT^ or SU-DHL4-shGNA13^UTR^-OE^C14/18S^ cells into recipient NOD/SCID mice. Tertiary recipients transplanted with two different SU-DHL4 cells were randomly assigned to two cohorts that were orally administered 100 mg/kg ABT-199 or vehicle, respectively. As shown in Fig. 5B, SU-DHL4-shGNA13^UTR^-OE^C14/18S^ tumors in mice treated with vehicle control (*n* = 13) grew faster than SU-DHL4-shGNA13^UTR^-OE^WT^ tumors (*n* = 13), which is consistent with previous in vitro result in Fig. 2D. The ABT-199 treatment slowed down the growth of shGNA13^UTR^-OE^WT^ tumors (*n* = 10), while the effect of the ABT-199 treatment was much more dramatic on SU-DHL4-shGNA13^UTR^-OE^C14/18S^ tumors (*n* = 8) (Fig. 5B). At the end of this experiment (56 days), 8 out of 8 SU-DHL4-shGNA13^UTR^-OE^C14/18S^ mice (100%) treated with the ABT-199 remained tumor-free. These data suggest that inhibition of palmitoylation of GNA13 is an effective therapeutic strategy for GCB-DLBCL in combination with the BCL2 inhibitor.

## Discussion

In this study, we demonstrate that palmitoylation of GNA13 not only regulates its plasma membrane localization, but also regulates GNA13’s stability. It is essential for the tumor suppressor function of GNA13 in GCB-DLBCL cells. Interestingly, we found that GNA13 negatively regulates BCL2 expression in GCB-DLBCL in a palmitoylation-dependent manner. Consistently, we found that GCB-DLBCL with loss-of-function mutations of GNA13 are sensitive to the treatment with BCL2 inhibitors and that inactivating the WT GNA13 by targeting its palmitoylation dramatically enhanced the sensitivity of GCB-DLBCL to the BCL2 inhibitor. These results indicate that the loss-of-function mutation of GNA13 is a biomarker for the precision BCL2 inhibitor therapy for GCB-DLBCL and that GNA13 palmitoylation is a potential target for combination therapy with the BCL2 inhibitor to treat GCB-DLBCL with WT GNA13.

It has been shown that palmitoylation regulates protein stability^26^. We show here that palmitoylation of GNA13 does affect its stability (Fig. 1B). Our data show that downregulation of palmitoylation-deficient mutant GNA13^C14/18S^ was rescued significantly by caspase inhibitors such as Z-VAD(OMe)-FMK (Fig. 1D), indicating that palmitoylation of GNA13 regulates its stability by inhibiting caspase-mediated protein degradation. It is possible that palmitoylation affects the conformation of GNA13, exposing the caspase cleavage sites. Alternatively, changed cellular localization due to lack of palmitoylation may subject GNA13 to the caspase-mediated protein degradation. These possibilities will be tested in the future.

Mutations of *GNA13* have been found in about 18% in GCB-DLBCL, 13% Burkitt lymphoma (BL), and 15.6% Follicular Lymphoma (FL), respectively^43–45^, including multiple point mutations and truncated variants. In BL, several prevailing GNA13 point mutations, such as L184R, L197Q, and F245S, were shown to be loss-of-function mutations in terms of Gα13/RHOA activity in GPCR signaling^46^. Here, we identified two palmitoylation sites, C14 and C18, in the N-terminal GNA13 protein by using both genetic and biochemical approaches. Consistent with our finding that palmitoylation of GNA13 is required for its tumor suppressor function, at least two DLBCL patients were found to bear the GNA13 C14S mutation^38,39^. Our data further support the idea that GNA13 plays tumor suppressor function in germinal center B cells and loss-of-function mutations of GNA13 are involved in lymphomagenesis.

Venetoclax, the FDA-approved BCL2 inhibitor^47,48^, is being tested to extend its application in DLBCL^49^. Our study indicates that inactivation of GNA13 alone could lead to higher expression of BCL2 that eventually conferred higher drug sensitivity to BCL2 inhibitor, implying that DLBCL patients with inactive mutations of GNA13 are more sensitive to the BCL2 inhibitor treatment, providing a patient-stratification biomarker for the venetoclax therapy.

Inactivating a tumor suppressor may sound counterintuitive in fighting cancers. But it works as a strategy of synthetic lethality. A recent systematic drug sensitivity screening shows that loss of SETD2 sensitized tumor cells to CDK7 inhibitor, and BAP1 depletion confers vulnerability to inhibitor of DNMT1^50^. Our work that inactivating GNA13 increases sensitivity of GCB-DLBCL cells to the BCL2 inhibitor may serve as a new therapeutic strategy for GCB-DLBCL.

S-palmitoylation is catalyzed by PATs. To date, at least 23 mammalian PATs have been identified^51^. The limited responsibilities of each PAT give hope that targeting specific PAT may be safe and effective in cancer therapies. It is important to identify which PAT is responsible for the palmitoylation modification of GNA13 and develop specific PAT inhibitors. Such agents would be effective for treating GCB-DLBCL with WT GNA13 in combination with the BCL2 inhibitor.

As mentioned in the Introduction, the role of GNA13 in tumorigenesis is cell-context dependent. It is not clear whether GNA13 also regulates the expression of BCL2 in tumors other than GCB-DLBCL and whether the therapeutic scenario for targeting palmitoylation of GNA13 in combination with the BCL2 inhibitor holds in other types of cancer. We will test the role of palmitoylation of GNA13 in other type of cancer in the future.

## Materials and methods

### Cell culture

The GCB-DLBCL cell lines SU-DHL6 and OCI-LY1 were kindly provided by Dr. Chenghua Yang, Shanghai Institute of Nutrition and Health, Chinese Academy of Sciences (CAS). The GCB-DLBCL cell line SU-DHL4 was obtained from Stem Cell Bank, CAS. All the GCB-DLBCL cell lines were cultured in RPMI 1640 (Basal Media, Shanghai, China) with 10% (v/v) FBS (Thermo, Waltham, MA, USA) in a humidified incubator at 37 °C under 5% CO_2_. The HeLa cells were bought from ATCC and cultured as previously described^52^. All cell lines were authenticated via STR profiling and periodically treated with Plasmocin (Invivogen, San Diego, CA, USA) to exclude mycoplasma contamination.

### Cell proliferation/viability assay

Cell proliferation/viability were assessed using CellTiter-Glo Luminescent Cell Viability Assay (Promega, Madison, WI, USA) as previously described^52,53^. Briefly, cells were seeded into 96-well cell plates (5000 cells/well) and supplemented with drugs at various concentrations. After 48 h of incubation, cells were lysed by CellTiter-Glo reagent and the resulting luminescence was measured using an Envision plate reader (PerkinElmer, Akron, OH, USA) after a 30-min incubation at room temperature.

### Chemicals and antibodies

The chemical library containing FDA-approved drugs and bioactive compounds, BCL2 inhibitor navitoclax (ABT-263), AKT inhibitor MK-2206, and proteasome inhibitor MG-132 were purchased from Selleckchem (Houston, TX, USA). The BCL2 inhibitor venetoclax (ABT-199), the pan-caspase inhibitor Emricasan and Q-VD-Oph, the group II caspases inhibitor Ac-DEVD-CHO, the pan-PI3K inhibitor GDC-0941, and the PI3Kα/δ inhibitor Copanlisib were purchased from CSNpharm (Arlington Heights, IL, USA). The cycloheximide (CHX), autophagy inhibitors, Hydroxychloroquine sulfate (HCQ), ULK-101, and the pan-caspase inhibitor Z-VAD(OMe)-FMK were purchased from TargetMol (Wellesley Hills, MA, USA). For antibodies, the rabbit anti-GNA13 (ab128900), rabbit anti-Na-K-ATPase (ab76020), and rabbit anti-BCL2 (ab32124) antibodies were purchased from Abcam (Cambridge, UK). The rabbit anti-Caspase3 (9662), rabbit anti-phospho-AKT (Ser473(4060)), rabbit anti-pan-AKT (4691), rabbit anti-HA (3724), anti-Rabbit IgG (H + L), and F(ab′)2 Fragment (Alexa Fluor^®^ 555 Conjugate) (4413) antibodies were purchased from Cell Signaling (Danvers, MA, USA). The HRP-conjugated mouse anti-GAPDH (HRP-60004) and HRP-conjugated mouse anti-α-Tubulin (HRP-66031) antibodies were bought from Proteintech (Rosemont, IL, USA). The goat anti-Biotin (B 3604) was purchased from Sigma Aldrich (St. Louis, MO, USA). The donkey anti-Goat IgG (H + L) Cross-Adsorbed Secondary Antibody, Alexa Fluor 546 (A-11056) was bought from Thermo.

### Membrane and cytosolic protein isolation

Protein extractions from membrane and cytosolic part of cells were isolated as previously described^54,55^.

### Western blot analysis

Western blot analysis was performed as previously described^53^. Briefly, cells were lysed in 1× sodium dodecyl sulfate (SDS) sample loading buffer, and then equal amounts of protein samples were loaded to polyacrylamide gel, transferred to nitrocellulose membrane, and then blotted with specific primary and secondary antibodies. Luminescence signals on membrane were detected with Immobilon Western HRP Substrate (Millipore, Darmstadt, Germany) and blots were imaged by the FluorChem Multiplex imaging system (ProteinSimple, San Jose, CA, USA).

### Drug screening

Both SU-DHL4-Scr and SU-DHL4-UTR cells were seeded into 96-well plates at a density of 5000 cells/well. Individual chemicals from FDA-approved Drug Library were added into each well in duplicates by an Explorer automation workstation (Perkin Elmer) at the concentration of 2 μM. After 48 h of incubation, cell viability was measured using CellTiter-Glo (Promega) following the manufacturer’s instructions.

### Statistical analysis

GraphPad Prism 7 and the Student’s *t*-test were used for data analysis. Statistical significance threshold was set at *P* = 0.05, and different levels were denoted as *, *P* < 0.05, **, *P* < 0.01, and ***, *P* < 0.001, respectively.

## Supplementary information

Supplementary Figure 1

Supplementary Figure 2

Supplementary Figure 3

Supplementary Figure 4

Supplementary Figure 5

Supplementary Figure Legends

Supplemental Material

## References

[1] Kelly P, Casey PJ, Meigs TE (2007). Biologic functions of the G12 subfamily of heterotrimeric G proteins: growth, migration, and metastasis. Biochemistry.

[2] Muppidi JR (2014). Loss of signalling via Gα_13_ in germinal centre B-cell-derived lymphoma. Nature.

[3] Ruppel KM (2005). Essential role for Gα_13_ in endothelial cells during embryonic development. Proc. Natl Acad. Sci. USA.

[4] Sivaraj KK (2013). G13 controls angiogenesis through regulation of VEGFR-2 expression. Dev. Cell.

[5] Chen L, Zhang JJ, Rafii S, Huang XY (2009). Suppression of tumor angiogenesis by Gα(13) haploinsufficiency. J. Biol. Chem..

[6] Wu M (2017). Gα13 negatively controls osteoclastogenesis through inhibition of the Akt–GSK3β–NFATc1 signalling pathway. Nat. Commun..

[7] Moers A (2003). G13 is an essential mediator of platelet activation in hemostasis and thrombosis. Nat. Med..

[8] Shen B (2013). A directional switch of integrin signalling and a new anti-thrombotic strategy. Nature.

[9] Mu G (2018). Gastrin stimulates pancreatic cancer cell directional migration by activating the Gα12/13–RhoA–ROCK signaling pathway. Exp. Mol. Med..

[10] Rasheed SA, Teo CR, Beillard EJ, Voorhoeve PM, Casey PJ (2013). MicroRNA-182 and microRNA-200a control G-protein subunit α-13 (GNA13) expression and cell invasion synergistically in prostate cancer cells. J. Biol. Chem..

[11] Yagi H (2016). GEP oncogene promotes cell proliferation through YAP activation in ovarian cancer. Oncogene.

[12] Morin RD (2013). Mutational and structural analysis of diffuse large B-cell lymphoma using whole-genome sequencing. Blood.

[13] Schmitz R (2018). Genetics and pathogenesis of diffuse large B-cell lymphoma. N. Engl. J. Med..

[14] Sha C (2019). Molecular high-grade B-cell lymphoma: defining a poor-risk group that requires different approaches to therapy. J. Clin. Oncol..

[15] Miao Y, Medeiros LJ, Li Y, Li J, Young KH (2019). Genetic alterations and their clinical implications in DLBCL. Nat. Rev. Clin. Oncol..

[16] Alizadeh AA (2000). Distinct types of diffuse large B-cell lymphoma identified by gene expression profiling. Nature.

[17] Swerdlow SH (2016). The 2016 revision of the World Health Organization classification of lymphoid neoplasms. Blood.

[18] Liu Y (2012). S1PR1 is an effective target to block STAT3 signaling in activated B cell-like diffuse large B-cell lymphoma. Blood.

[19] Muppidi JR, Lu E, Cyster JG (2015). The G protein-coupled receptor P2RY8 and follicular dendritic cells promote germinal center confinement of B cells, whereas S1PR3 can contribute to their dissemination. J. Exp. Med..

[20] Baldari CT (2016). S1PR2 deficiency in DLBCL: a FOXy connection. Blood.

[21] O’Hayre M (2013). The emerging mutational landscape of G proteins and G-protein-coupled receptors in cancer. Nat. Rev. Cancer.

[22] Caeser R (2019). Genetic modification of primary human B cells to model high-grade lymphoma. Nat. Commun..

[23] Cattoretti G (2009). Targeted disruption of the S1P2 sphingosine 1-phosphate receptor gene leads to diffuse large B-cell lymphoma formation. Cancer Res..

[24] Lohr JG (2012). Discovery and prioritization of somatic mutations in diffuse large B-cell lymphoma (DLBCL) by whole-exome sequencing. Proc. Natl Acad. Sci. USA.

[25] Reddy A (2017). Genetic and functional drivers of diffuse large B cell lymphoma. Cell.

[26] Ko PJ, Dixon SJ (2018). Protein palmitoylation and cancer. EMBO Rep..

[27] Linder ME (1993). Lipid modifications of G proteins: alpha subunits are palmitoylated. Proc. Natl Acad. Sci. USA.

[28] Cuiffo B, Ren R (2010). Palmitoylation of oncogenic NRAS is essential for leukemogenesis. Blood.

[29] Bhattacharyya R, Wedegaertner PB (2000). Gα_13_ requires palmitoylation for plasma membrane localization, rho-dependent signaling, and promotion of p115-rhoGEF membrane binding. J. Biol. Chem..

[30] Ren J (2008). CSS-Palm 2.0: an updated software for palmitoylation sites prediction. Protein Eng. Des. Sel..

[31] Weng SL, Kao HJ, Huang CH, Lee TY (2017). MDD-Palm: identification of protein S-palmitoylation sites with substrate motifs based on maximal dependence decomposition. PLoS ONE.

[32] Varshavsky A (2017). The ubiquitin system, autophagy, and regulated protein degradation. Annu Rev. Biochem.

[33] Senft D, Qi J, Ronai ZA (2018). Ubiquitin ligases in oncogenic transformation and cancer therapy. Nat. Rev. Cancer.

[34] Levy JMM, Towers CG, Thorburn A (2017). Targeting autophagy in cancer. Nat. Rev. Cancer.

[35] Salvesen GS, Dixit VM (1997). Caspases: intracellular signaling by proteolysis. Cell.

[36] Julien O, Wells JA (2017). Caspases and their substrates. Cell Death Differ..

[37] Zhou X (2016). Exploring genomic alteration in pediatric cancer using ProteinPaint. Nat. Genet.

[38] Mareschal S (2016). Whole exome sequencing of relapsed/refractory patients expands the repertoire of somatic mutations in diffuse large B-cell lymphoma. Genes Chromosomes Cancer.

[39] Zhou Y (2018). Analysis of genomic alteration in primary central nervous system lymphoma and the expression of some related genes. Neoplasia.

[40] Antonov AV (2014). PPISURV: a novel bioinformatics tool for uncovering the hidden role of specific genes in cancer survival outcome. Oncogene.

[41] Souers AJ (2013). ABT-199, a potent and selective BCL-2 inhibitor, achieves antitumor activity while sparing platelets. Nat. Med.

[42] Bojarczuk K (2019). Targeted inhibition of PI3Kα/δ is synergistic with BCL-2 blockade in genetically defined subtypes of DLBCL. Blood.

[43] Dubois S (2016). Next-generation sequencing in diffuse large B-cell lymphoma highlights molecular divergence and therapeutic opportunities: a LYSA study. Clin. Cancer Res..

[44] Wagener R (2019). The mutational landscape of Burkitt-like lymphoma with 11q aberration is distinct from that of Burkitt lymphoma. Blood.

[45] Shimono J (2018). Analysis of GNA13 protein in follicular lymphoma and its association with poor prognosis. Am. J. Surgical Pathol..

[46] O’Hayre M (2016). Inactivating mutations in GNA13 and RHOA in Burkitt’s lymphoma and diffuse large B-cell lymphoma: a tumor suppressor function for the Gα13/RhoA axis in B cells. Oncogene.

[47] Jain N (2019). Ibrutinib and venetoclax for first-line treatment of CLL. N. Engl. J. Med..

[48] Delbridge ARD, Grabow S, Strasser A, Vaux DL (2016). Thirty years of BCL-2: translating cell death discoveries into novel cancer therapies. Nat. Rev. Cancer.

[49] Zhao X (2019). BCL2 amplicon loss and transcriptional remodeling drives ABT-199 resistance in B cell lymphoma models. Cancer Cell.

[50] Ding H (2019). Systematic analysis of drug vulnerabilities conferred by tumor suppressor loss. Cell Rep..

[51] Greaves J, Chamberlain LH (2011). DHHC palmitoyl transferases: substrate interactions and (patho)physiology. Trends Biochemical Sci..

[52] Ning N (2018). A novel microtubule inhibitor overcomes multidrug resistance in tumors. Cancer Res.

[53] Wu M (2017). N-arachidonoyl dopamine inhibits NRAS neoplastic transformation by suppressing its plasma membrane translocation. Mol. Cancer Ther..

[54] Zhuang L, Lin J, Lu ML, Solomon KR, Freeman MR (2002). Cholesterol-rich lipid rafts mediate akt-regulated survival in prostate cancer cells. Cancer Res..

[55] Adam RM, Yang W, Di Vizio D, Mukhopadhyay NK, Steen H (2008). Rapid preparation of nuclei-depleted detergent-resistant membrane fractions suitable for proteomics analysis. BMC Cell Biol..

