# Supplementary figures and images for: GNA13 regulates BCL2 expression and the sensitivity of GCB-DLBCL cells to BCL2 inhibitors in a palmitoylation-dependent manner
Source: Cell Death Dis. 2021 Jan 9;12(1):54. doi: 10.1038/s41419-020-03311-1 (PMC7797003; doi:10.1038/s41419-020-03311-1)

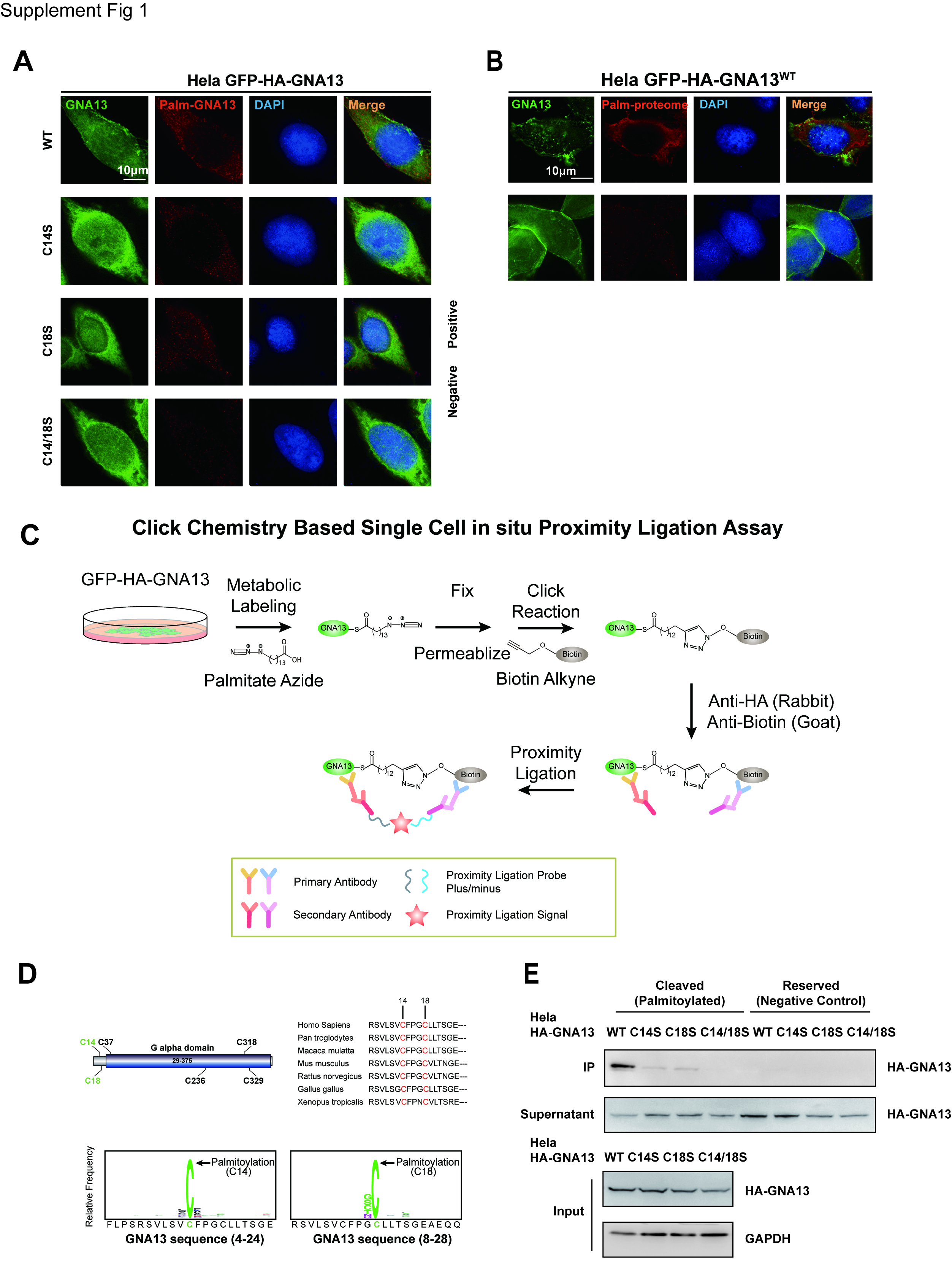

Supplement: Supplementary file 1 — Supplementary Figure 1 [file 41419_2020_3311_MOESM1_ESM.tif]

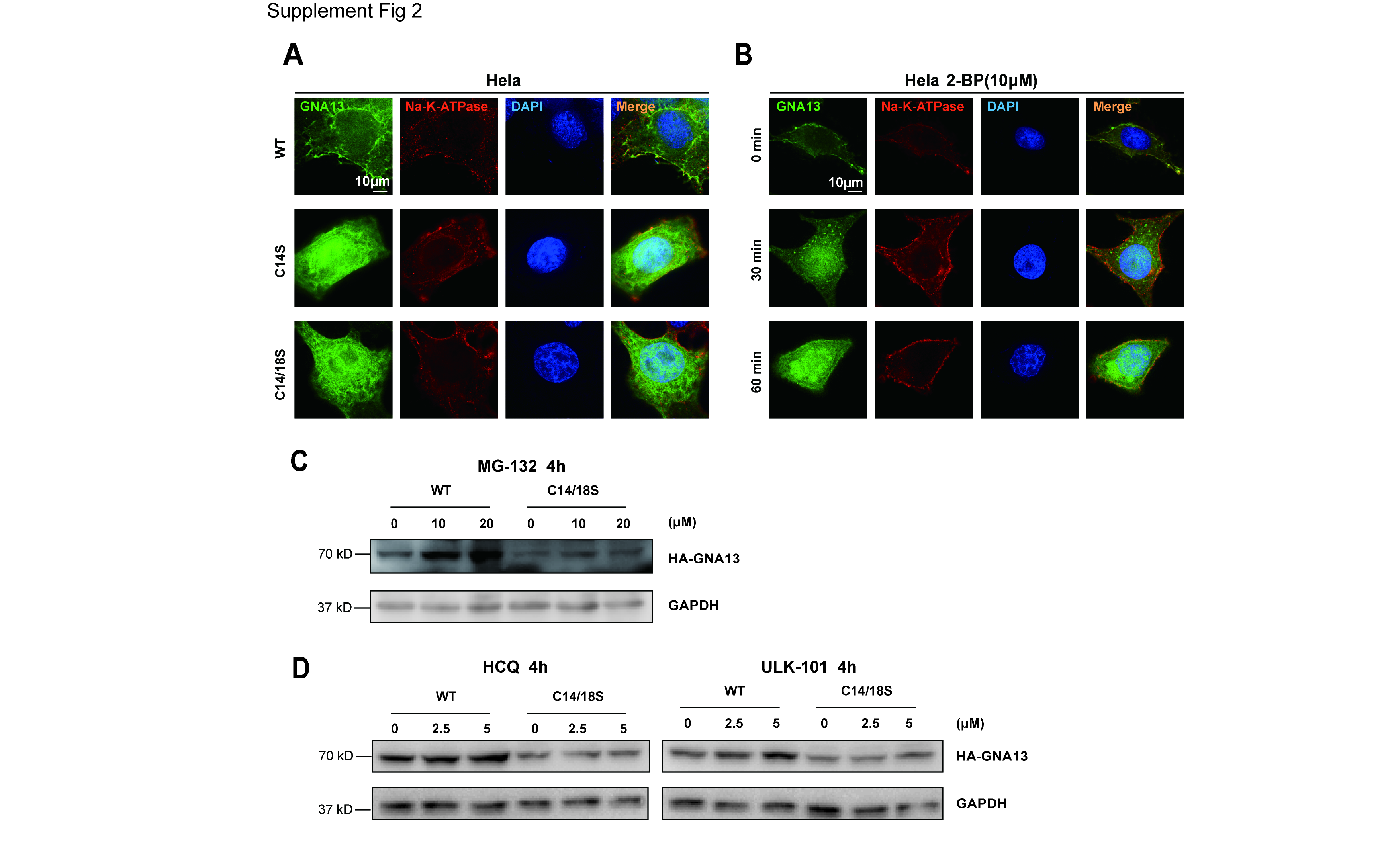

Supplement: Supplementary file 2 — Supplementary Figure 2 [file 41419_2020_3311_MOESM2_ESM.tif]

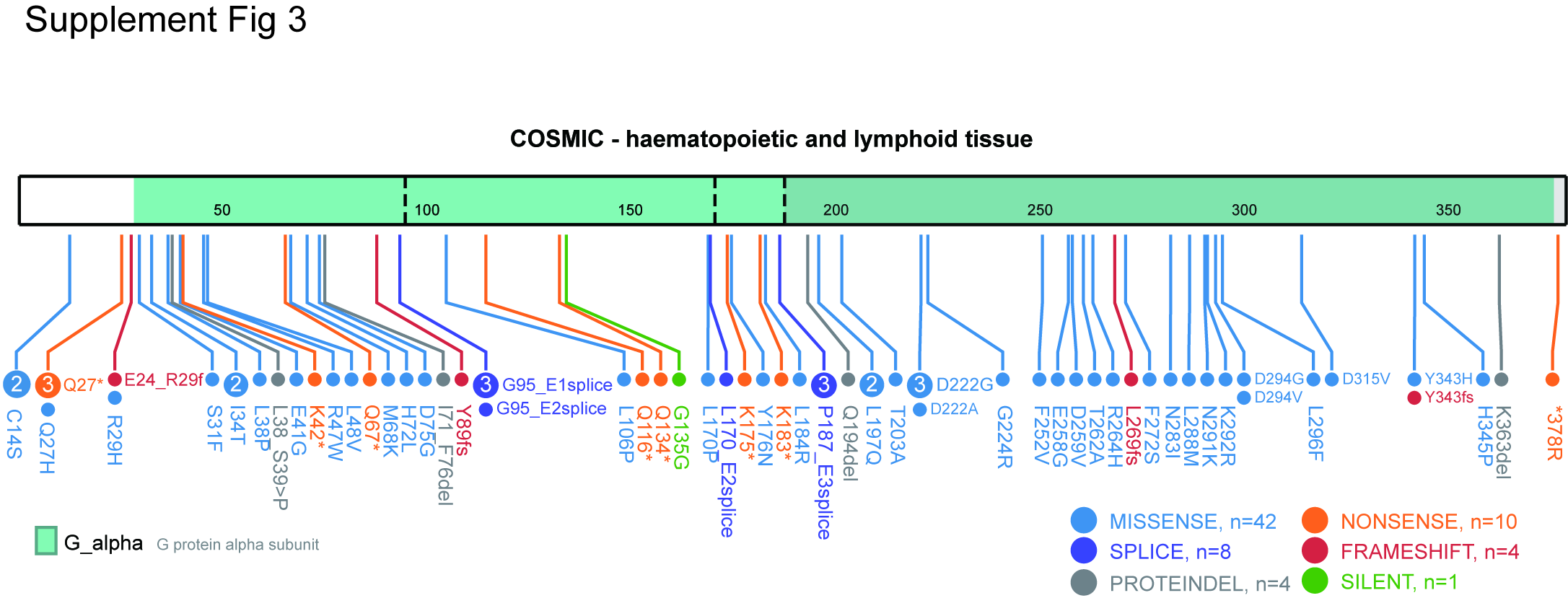

Supplement: Supplementary file 3 — Supplementary Figure 3 [file 41419_2020_3311_MOESM3_ESM.tif]

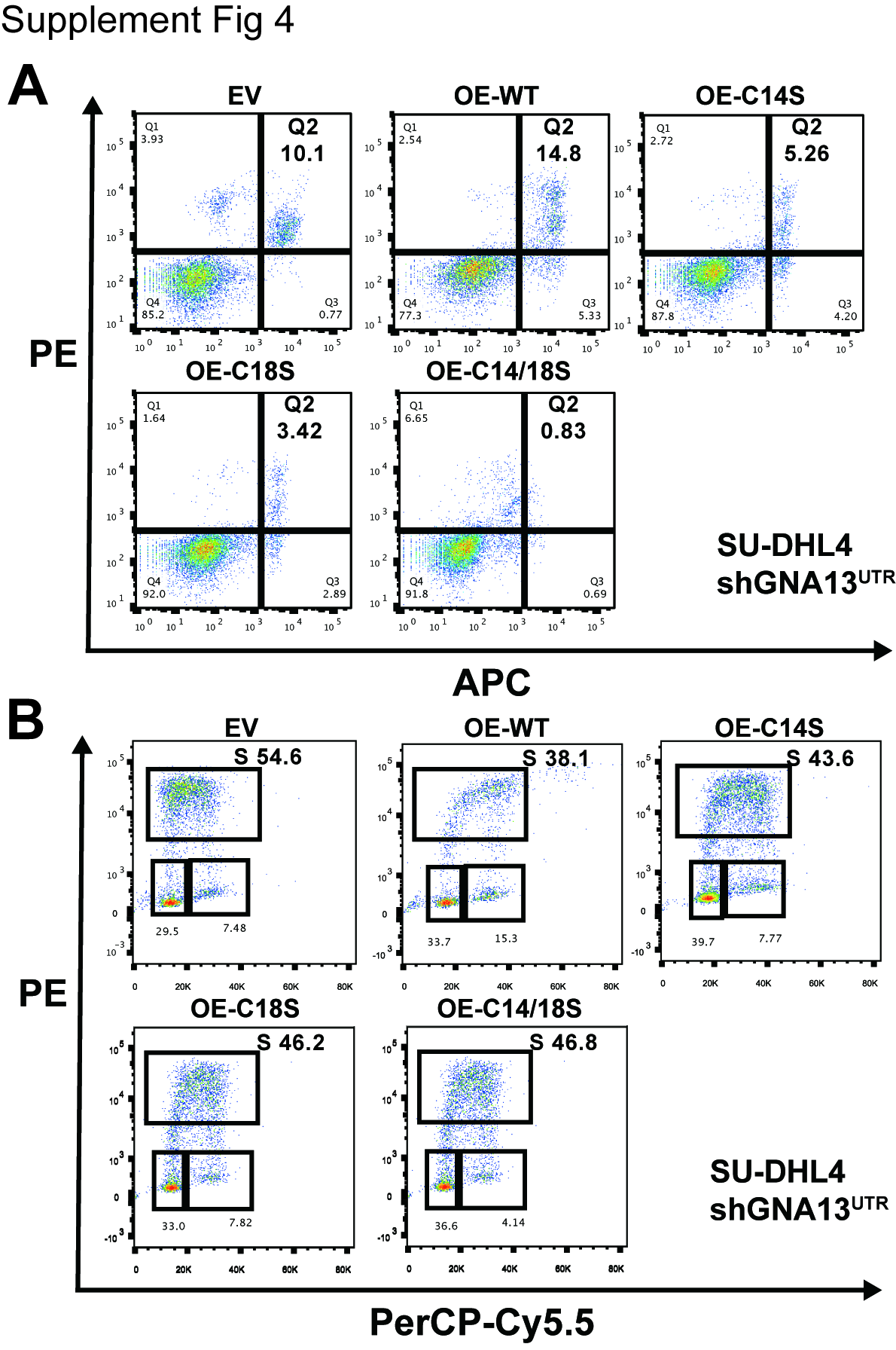

Supplement: Supplementary file 4 — Supplementary Figure 4 [file 41419_2020_3311_MOESM4_ESM.tif]

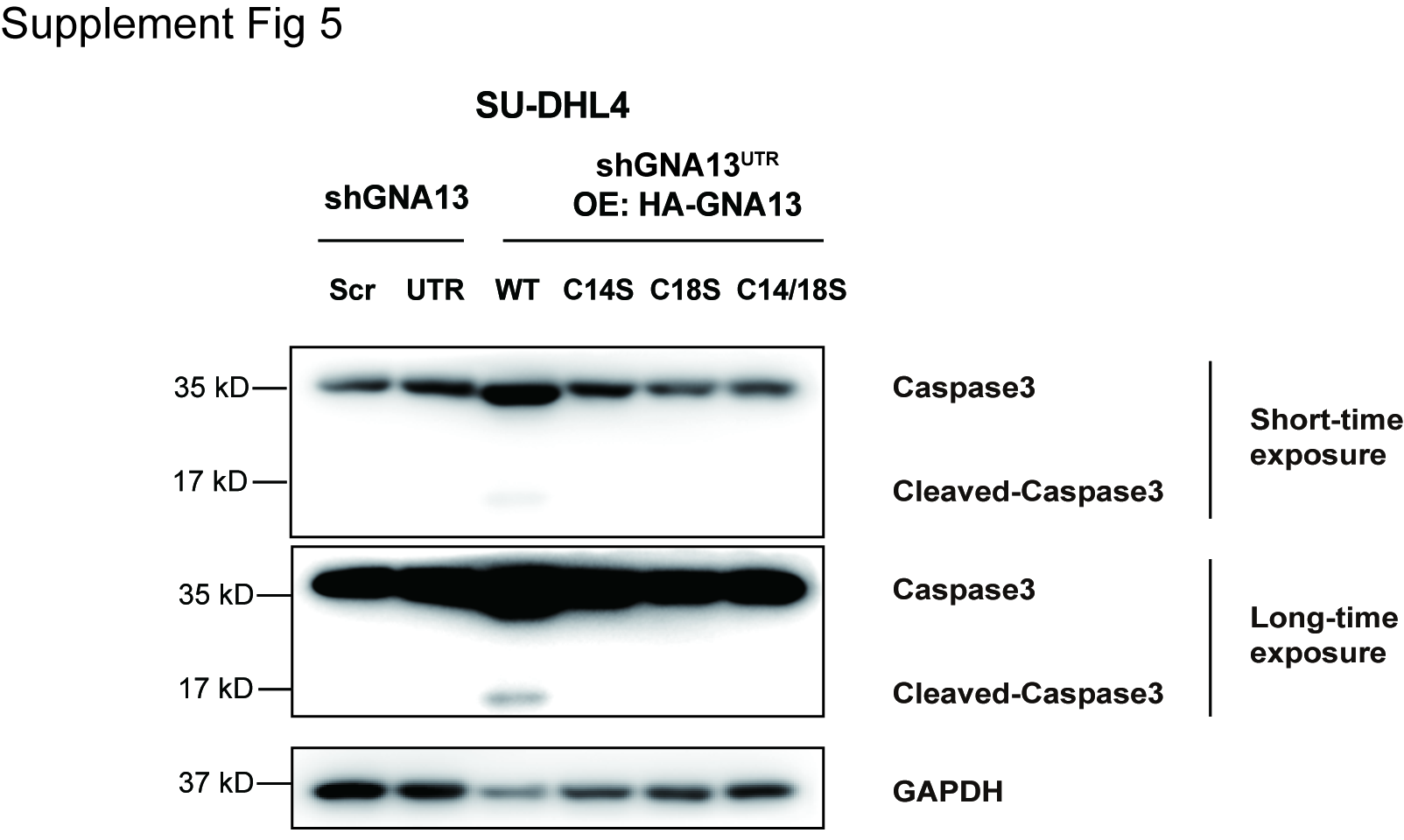

Supplement: Supplementary file 5 — Supplementary Figure 5 [file 41419_2020_3311_MOESM5_ESM.tif]
