# Supplementary material for: GNA13 regulates BCL2 expression and the sensitivity of GCB-DLBCL cells to BCL2 inhibitors in a palmitoylation-dependent manner
Source: Cell Death Dis. 2021 Jan 9;12(1):54. doi: 10.1038/s41419-020-03311-1 (PMC7797003; doi:10.1038/s41419-020-03311-1)
Supplement: Supplementary file 6 — Supplementary Figure Legends [file 41419_2020_3311_MOESM6_ESM.docx]

**Supplementary Figure** **Legends**

**Supplementary Figure 1.** **Detecting** **palmitoylated GNA13 by both PLA and Acyl-RAC assay.** (A) Detection of palmitoylated GNA13 (red) in HeLa cells transfected with the indicated constructs using click chemistry based single cell in situ proximity ligation assay (PLA). GFP-fused GNA13 (green) and DAPI staining (blue) are shown. Scale bar, 10 μm. (B) To reveal total protein palmitoylation, namely palmitoylation proteome (palm-proteome), cells were incubated with goat anti-biotin antibody and donkey anti-goat IgG Alexa Fluor 546 antibody after click chemistry reaction. Cells without labeling palmitate azide were used as negative controls. Scale bar, 10 μm. (C) Scheme of click chemistry-based single cell in situ proximity ligation assay of palmitoylated GNA13^1^. Hela cells that stably expressing GFP-HA-GNA13 were metabolically labeled with palmitate azide. After fixation and permeabilization, cells were labeled with a biotin alkyne by Huisgen cycloaddition reaction. The cells were then incubated with primary antibodies (rabbit anti-HA and goat anti-biotin antibodies) followed by proximity ligation assay to reveal the palmitoylated GNA13. (D) Analysis of palmitoylation sites in GNA13 using the CSS-palm 4.0 (upper left) and MDD-PALM (lower pane) palmitoylation sites prediction algorithms^2,3^. Upper right, alignment of sequences of GNA13 family proteins, including human, chimpanzee, rhesus macaque, mouse, rat, chicken and xenopus. (E) Acyl-RAC assays of HeLa cells transfected with the indicated constructs. The wildtype (WT) GNA13 is shown to be palmitoylated (cleaved fraction of immunoprecipitants), while palmitoylation of GNA13 C14S and C18S mutants are dramatically decreased. Palmitoylation of GNA13 C14/18S mutant is undetectable. The preserved fraction of immunoprecipitants show the specificity of the assay. The cleaved fraction and preserved fraction of supernatants represent non-palmitoylated proteins and the total protein unbound to the resin, respectively.

**Supplementary Figure 2.** **Palmitoylation of GNA13 regulates its plasma membrane association and stability.** (A)Representative images of localization of GFP-fused GNA13 on HeLa cells stably expressing WT GNA13, C14S, or C14/18S mutant of GNA13. Cells were stained with an anti-Na-K-ATPase antibody (red) as a plasma membrane marker and DAPI (blue). Scale bars, 10 μm. (B) Representative images of localization of GFP-fused WT GNA13 in HeLa cells before and after treatment of 10 μM 2-BP for 30 or 60 minutes. (C-D) Levels of WT GNA13 and C14/18S mutant of GNA13 in HeLa cells with or without the treatment of inhibitors for (C) proteasome (MG-132) and (D) autophagy-lysosome system (HCQ and ULK-101) were detected by immunoblotting with an anti-HA antibody. α-Tubulin and GAPDH were used as loading controls.

**Supplementary Figure 3.** **Mutation sites of GNA13 in DLBCL.** Schematic presentation of GNA13 protein domain and mutations in DLBCL. Whole genome sequencing data for relapse tumor samples were obtained from St. Jude Cloud (<https://www.stjude.cloud>).

**Supplementary Figure 4. Representative flowcytometry analysis of Annexin V/PI apoptosis assay and BrdU cell cycle progression assay.** (A-B) SU-DHL4 shGNA13^UTR^ cells were transfected with constructs containing empty vector (EV), GNA13^WT^, GNA13^C14S^, GNA13^C18S^ and GNA13^C14/18S^. (A) Apoptosis of each cell line was examined by Annexin V/PI flowcytometry assay 48 hours after plating. (B) The cell-cycle progression using a BrdU/7-AAD labeling assay was employed to assess proliferation of cells with different GNA13 palmitoylation-blocked constructions.

**Supplementary Figure 5. The status of Caspase-3 and cleaved Caspase-3 in different genetic backgrounds of SU-DHL4 cells.** After knock down endogenous GNA13 in SU-DHL4 with shRNA, we introduced GNA13^WT^ and several palmitoylated-block mutants of GNA13 into the SU-DHL4 shGNA13^UTR^. The cleaved Caspase3 was higher in cell overexpressed GNA13^WT^ than any other cells. The long-time exposure figure could make it clear.

**References**

1 Gao, X. & Hannoush, R. N. Single-cell imaging of Wnt palmitoylation by the acyltransferase porcupine. *Nat Chem Biol* **10**, 61-68 (2014).

2 Ren, J. *et al.* CSS-Palm 2.0: an updated software for palmitoylation sites prediction. *Protein Eng Des Sel* **21**, 639-644 (2008).

3 Weng, S. L., Kao, H. J., Huang, C. H. & Lee, T. Y. MDD-Palm: Identification of protein S-palmitoylation sites with substrate motifs based on maximal dependence decomposition. *PLoS One* **12**, e0179529 (2017).
