# Supplementary material for: GNA13 regulates BCL2 expression and the sensitivity of GCB-DLBCL cells to BCL2 inhibitors in a palmitoylation-dependent manner
Source: Cell Death Dis. 2021 Jan 9;12(1):54. doi: 10.1038/s41419-020-03311-1 (PMC7797003; doi:10.1038/s41419-020-03311-1)
Supplement: Supplementary file 7 — Supplemental Material [file 41419_2020_3311_MOESM7_ESM.docx]

**Supplementary Materials and Methods**

**Plasmid construction**

The shRNAs against human *GNA13* were ordered from Gene Pharma Corporation (Shanghai, China). DNA encoding the *GNA13 or* scrambled shRNA were cloned into pLKO.1 (Addgene #10878).

Scrambled sequence: TTCTCCGAACGTGTCACGTTTCTCGAGAAACGTGACACGTTCGGAGAA

shGNA13-600: GGCATCCATGAATACGAC

shGNA13-UTR: TAGCAGTTTACAACCAGAA

The construct expressing shRNA-resistant *GFP-HA-GNA13* was cloned into a mammalian expression vector LentiCas9-Blast (Addgene #52962), in which the sequence of Cas9 was removed by BamHI and XbaI restriction sites. The GNA13^C14S^, GNA13^C18S^, GNA13^C14/18S^ mutants were generated by site-directed mutagenesis using Mut Express^®^II Fast Mutagenesis Kit V2 (Vazyme, Nanjing, China) per manufacturer’s instruction. All constructs were verified by Sanger sequencing via Sunnybio (Shanghai, China).

**Lentivirus production and transduction**

Lentiviruses were generated following the protocols from Addgene^1^. Briefly, a 6 cm dish of 80% confluent HEK293T cells were transfected with 1 μg pLKO.1 containing shRNA sequences, 750 ng psPAX2 packaging plasmid DNA, 250 ng pMD2.G envelope plasmid DNA, 500 µL serum-free Opti-MEM^®^ Medium and 10 μL Lipo6000^TM^ (Beyotime, Shanghai, China) transfection reagent. Forty-eight hours post transfection, viral supernatants were collected and filtered through a 0.22 μm low protein binding membrane (Millipore, Darmstadt, Germany). After SU-DHL4 cells were seeded in 6-well plates, 1 mL viral supernatant with 8 µg/mL polybrene (Sigma, St. Louis, MO, USA) was added to cells. This was followed by centrifugation at 2000 rpm for 90 min at room temperature. Forty-eight hours later, the target cells were cultured in the presence of 2 μg/mL puromycin.

**Apoptotic assay**

Cell apoptosis was examined using eBioscience™ Annexin V Apoptosis Detection Kit APC (Thermo, Waltham, MA, USA) per manufacturer’s instruction. Flow cytometry data were analyzed via FlowJo software.

**BrdU labeling assay**

Cells were incubated with 10 μM of BrdU for 2 h, fixed with Cytofix/Cytoperm Buffer (BD), and incubated for 1 h with DNase at 37 ̊C. PE-conjugated anti-BrdU antibody (BD) was then added for 30min at room temperature. Then cells were washed and DNA was stained using 7-AAD 5 μL per sample (BD) followed by flow cytometry analysis, thus the cell in S phase could be labeled with BrdU.

**Isobaric iodoTMT switch labeling-based mass spectrometry assay**

HeLa cells expressing HA-GNA13 were rinsed three times with 4°C pre-cold phosphate-buffered saline (PBS) and lysed by 1 mL lysis buffer (LB) (50 mM Tris-HCl, pH 7.4, 150 mM NaCl, 1 mM EDTA, 1% Triton X-100) with 10mM Tris (2-carboxyethyl) phosphine hydrochloride (TCEP, Sigma Aldrich), 50 mM N-Ethylmaleimide (NEM, Sigma Aldrich), 1× protease inhibitor cocktail (Roche, Basel, CH) and 1× Phenylmethylsulphonyl fluoride (PMSF, Beyotime) at 4°C for 2 hours with constant rotation. Excess NEM was removed by the Zeba desalt spin column (Thermo). After centrifugation at maximum speed for 20 minutes at 4 °C, the supernatant was incubated for overnight with 50 μL pre-washed anti-HA agarose (Sigma Aldrich, St. Louis, MO, USA). The beads were then equally divided into two parts and either treated with LB (-HAM group) or LB with 1M NH_2_OH (Sigma Aldrich) (+HAM group) for 2 hours at room temperature (RT). This was followed by iodoTMT labelling per manufacturer’s instruction. In brief, after on-beads denaturing with 6 M urea (Sigma Aldrich), the protein samples were incubated with iodoTMTsixplex reagents (Thermo) for 1 hour at 37°C under the dark condition. The reaction was quenched by adding 0.5 M DL-Dithiothreitol (DTT, Beyotime). Transfer equal amounts of each sample labeled with iodoTMTsixplex reagents into a separate tube. Elute samples with urea buffer (6 M urea, 1 mM EDTA, pH 8.2) for 3 times, followed by resuspension in urea buffer plus 5 x SDS sample buffer (250 mM Tris-HCl, 10% SDS, 50% glycerol, 50 mM DTT) at 100 °C for 10 minutes. The proteins were subjected to in-gel digestion and LC/MS analysis by the Proteomics Platform of Core Facility of Basic Medical Sciences, Shanghai Jiao Tong University School of Medicine (SJTU-SM).

**Acyl-Resin Assisted Capture (Acyl-RAC) Assay**

The level of palmitoylation was evaluated by the Acyl-RAC method using the CAPTUREome^TM^ S-Palmitoylated Protein Kit per manufacturer’s instruction (Badrilla, Leeds, UK) with minor optimization. Protein concentration of cell lysates was determined by Pierce BCA Protein Assay Kit (Thermo Fisher Scientific) after mild sonication. One milligram of protein was incubated in Thiol Block Buffer (pack components) for 4 hours at 40°C with 5 seconds vortexing every 20 minutes. After precipitation with ice-cold acetone, the protein pellets were disrupted and resuspended in 300 μL Binding Buffer (pack components) at 40°C for 2 hours. Subsequently, proteins were quantified by BCA Protein Assay. Fifty micrograms protein was taken out as input fraction (Input), and equal amounts of the rest protein sample were treated with Thioester Cleavage Reagent (experimental sample, namely Cleaved group) or Acyl Preservation Reagent (the negative control sample, namely Preserved group). Each sample was mixed with 50 μL CAPTUREome^TM^ Capture Resin and incubated for 3 hours at RT with end-over-end rotation. Fifty microliters of supernatant was kept and boiled with 2× Laemmli Sample Buffer for 10 minutes, referred as Supernatant sample. After stringent washes, resin was boiled at 60°C with 2× Laemmli Sample Buffer for 10 minutes. Captured proteins were separated by SDS-PAGE and analyzed by western blot.

**Click chemistry-based single cell *in situ* proximity ligation Assay**

To determine the level of palmitoylated GNA13 *in situ*, HeLa cells expressing either wildtype or mutant GFP-HA-GNA13 were subjected to a click chemistry-based proximity ligation assay (PLA) as previously reported^2^, with modifications. In brief, cells were seeded onto coverslips (Thermo). After 24 hours, Click-iT^®^ palmitic acid-azide (Thermo, Waltham, MA, USA) was added into culture medium of experimental samples, while palmitic acid (Sigma Aldrich) for negative control samples. Cells were incubated in a 5% CO_2_ humidified chamber for 18 h at 37 °C, and then fixed by adding 100 μL of prechilled methanol to each sample at −20 °C for 10 min. For each coverslip, prepare a 100 μL click reaction cocktail by sequentially adding 2 μL of Biotin alkyne (Thermo) solution (final concentration 0.1 mM), 2 μL of TCEP (Sigma Aldrich) solution (final concentration 0.1 mM) and 2 μL of CuSO_4_ (Sigma Aldrich) solution (final concentration 0.1 mM) in 94 μL of PBS. After incubated in blocking buffer for 1 h at room temperature, the samples were treated with anti-HA antibody (rabbit) overnight in the dark at 4 °C, followed by incubation with anti-Biotin antibody (goat) for 1 hour. The following steps of PLA were conducted per manufacturer’s instruction. The pair of Duolink^®^ In Situ PLA^®^ Probe Anti-Rabbit PLUS and Anti-Goat MINUS, the Detection Reagents Orange were purchased from Sigma Aldrich.

**Immunofluorescence imaging with super-resolution microscopy**

The coverslips from PLA experiments were visualized with a DeltaVision OMX super-resolution microscope using a 63× objective (GE Healthcare Life Sciences, Marlborough, MA, USA). Nuclei of cells were stained with DAPI or Hoechst 33342 and visualized under an ultraviolet laser with 436 filter. Plasma membrane was marked with specific antibody against to Na-K-ATPase and visualized with red fluorescence under a 555 nM laser with 609 filter. GFP fused GNA13 proteins were visualized under a 488 nM laser with 528 filter. The captured images were processed by NIH ImageJ software.

**Mouse GCB-DLBCL xenograft model and drug testing**

Female NOD/SCID mice (6-8-weeks-old) were purchased from Charles River Laboratories and fed in Shanghai Jiao Tong University School of Medicine Experimental Animal Center. All murine procedures were performed according to Shanghai Jiao Tong University Animal Care and Use Committee approved protocols. Three NOD/SCID mice were injected hypodermically with 5×10^6^ various GCB-DLBCL cells resuspended in 200 μL Matrigel (BD, Franklin Lakes, NJ, USA). When tumor grew large enough, the tumor tissues were surgically extracted and transplanted to new NOD/SCID mice as previously described^3^. This procedure was repeated on the secondary transplant to generate the tertiary NOD/SCID mice for the treatment experiment afterwards. ABT-199 was formulated in 60% phosal 50PG (MCE, Monmouth Junction, NJ, USA), 30% PEG400 (Sigma Aldrich) and 10% ethanol^4^. Seven days after tumor inoculation, tertiary NOD/SCID mice were grouped randomly and 100mg/kg ABT-199 or vehicle was orally administrated daily for 18 consecutive days. Experiments were stopped when the largest tumor grew up to 2000 mm^3^.

**References**

1 Moffat, J. *et al.* A lentiviral RNAi library for human and mouse genes applied to an arrayed viral high-content screen. *Cell* **124**, 1283-1298 (2006).

2 Gao, X. & Hannoush, R. N. Single-cell in situ imaging of palmitoylation in fatty-acylated proteins. *Nature Protocols* **9**, 2607-2623 (2014).

3 Ning, N. *et al.* A Novel Microtubule Inhibitor Overcomes Multidrug Resistance in Tumors. *Cancer Res* **78**, 5949-5957 (2018).

4 Wu, X. *et al.* Extra-mitochondrial prosurvival BCL-2 proteins regulate gene transcription by inhibiting the SUFU tumour suppressor. *Nature cell biology* **19**, 1226-1236 (2017).
